# Optical and structural characterization of femtosecond laser written micro-structures in germanate glass

**DOI:** 10.1038/s41598-023-35730-3

**Published:** 2023-07-08

**Authors:** Rayan Zaiter, Matthieu Lancry, Alexandre Fargues, Frédéric Adamietz, Marc Dussauze, Vincent Rodriguez, Bertrand Poumellec, Thierry Cardinal

**Affiliations:** 1 Institut de Chimie de la Matière Condensée de Bordeaux, Université de Bordeaux, 87 Avenue du Dr Schweitzer, 33608 Pessac, France; 2 Institut de Chimie Moléculaire et des Matériaux d’Orsay/SP2M/MAP, CNRS, Université Paris-Saclay, 91405 Orsay, France; 3 Institut des Sciences Moléculaires, UMR 5255, Université de Bordeaux, 351 Cours de la Libération, 33405 Talence Cedex, France

**Keywords:** Materials for optics, Glasses, Optics and photonics, Optical materials

## Abstract

We report on direct femtosecond laser writing in zinc barium gallo-germanate glasses. A combination of spectroscopic techniques allows to progress in the understanding of the mechanisms taking place depending on the energy. In the first regime (type I, isotropic local index change) up to 0.5 µJ, the main occurrence is the generation of charge traps inspected by luminescence, together with separation of charges detected by polarized second harmonic generation measurements. At higher pulse energies notably at the threshold corresponding to 0.8 µJ or in the second regime (type II modifications corresponding to nanograting formation energy domain), the main occurrence is a chemical change and re-organization of the network evidenced by the appearance of molecular O_2_ seen in the Raman spectra. In addition, the polarization dependence of the second harmonic generation in type II indicates that the organization of nanogratings may be perturbed by the laser-imprinted electric field.

Femtosecond Laser Direct Writing (FLDW) allows highly localized refractive index modifications with minimal side impact damages^1–4^. So far, no other manufacturing process has the potential to integrate 3D multifunctional components in a single monolithic chip and within a variety of transparent materials. Glasses such as SiO_2_ and GeO_2_ are two good glass model systems which have been used to investigate fs-laser-induced changes. Bressel et al. reported on structural modifications in GeO_2_ glass induced by tightly focused femtosecond laser beam^5–7^. In silicate glasses, a change in element distribution including network modifiers has been observed under high-repetition rate femtosecond laser irradiation^8^. These results indicate that an efficient driving force is the sharp temperature gradient, which originates from the thermal accumulation around the focal volume. However, at low-repetition rate depending on the laser regime, there is occurrence of nanogratings which consist of the self-assembly of nanostructures in the direction perpendicular to the light polarization^9^. Nanogratings possess several peculiar properties such as anisotropy light scattering, wavelength-dependent reflectivity and birefringence finding practical use in microfluidic channels^10^, light polarization converters^11^ and ultrastable 5D optical data storage^12^.

Silicate glasses are one of the most ubiquitous vitreous materials, known for their particular versatile platforms FLDW^13^, due to their commercial availability, excellent optical transparency and physico-chemical stability. Nonetheless, regarding the photonic applications, the use of silicate glasses are limited to the near infrared region (λ < 2 µm), and do not comply with the increasing demand for Mid-IR (up to 8 µm) applications requiring 3D laser manufacturing of miniaturized, low weight and low cost optical components. In turn, this will lead soon to their much awaited commercialization for various application fields not only for civilian (domotics, smartphone, automobile) but also for security and military applications including sensing of toxic gases, detection of explosives and countermeasure identification, as well as biophotonics (medicine) such as spectral tissue mapping for medical diagnosis^14^. Therefore, to access the above-mentioned Mid-IR range, non-silicate glass matrices must be used, such as chalcogenides^15^, fluorides^16^, or heavy metal oxides (HMO)^17^. Up to date, nanogratings formation has not been demonstrated in non-oxide glasses**.** Thus, among these potential optical materials, heavy metal oxide glasses (HMO), and more specifically barium gallo-germanate (BGG) glasses have emerged as potential candidates as they offer a combination of different properties^18^: a high rare-earth ions solubility, chemical stability, superior mechanical strength, a wide optical transparency extending up to ~ 6 µm in the Mid-IR as well as a fiber-shaping ability.

Recently, as classically observed in irradiated silicate glasses, different modification regimes were successfully reproduced in derived barium gallo-germanates depending on the laser pulse energy: Type I (permanent isotropic refractive index), Type II (birefringence attributed to self-assembled nanogratings) and spatial broadening regime (laser tracks become much larger than the beam size)^19^. Among the different studied derived BGGs, zinc barium gallo-germanate glass (8%ZnO-BGG) presented the highest birefringence indicating its high potential to be used for birefringence integrated optical applications^19^. On this basis, authors want to go further and understand better the different mechanisms occurring during the laser irradiation in this glass. For this reason, different techniques were employed, such as Raman spectroscopy, micro-luminescence and micro-second harmonic generation (SHG). These techniques are well-known to be pertinent for our purpose as structural analysis, SHG and luminescence in femtosecond laser structured materials can provide insights for the best compositional formulation of the glass and the best exposure conditions for nanograting formation. Luminescence induced by femtosecond laser irradiation has been already largely reported^20,21^, where defect centers are produced and have been claimed to be responsible for changes in the glass macroscopic properties such as density and refractive index. Numerical simulations show that they play an important role in driving self-organization of the nanograting structure, but so far the mechanism is still under debate^22^. Glass modifications are the result of relaxation of the photoinduced electrons. First, electrons relax into self-trapped excitons by electron–phonon coupling then are eliminated radiatively or not, or transform into point defects such as into SiE’ and non-bridging oxygen hole center (NBOHC) in the case of silica glass then into oxygen deficient centers SiODC(II)^20^. In the case of germanate glass, the photoinduced absorption peaks of the defect centers GeE’^23^ and NBOHC^24^ appear respectively at 400 and 650 nm.

Si et al*.* have reported that there is a correlation between the photo-induced SHG and the creation of a Ge electron center in germanosilicate glass^25^. Using electron-spin-resonance spectrometry, Tsai et al. have demonstrated that SHG is related to the formation of GeE’ center^26^. In other words, the stability of SHG signal depend on the type of photo-induced defects species, whereas the magnitude of SHG is linked to their concentration and distribution. Furthermore, Papon et al*.* have reported on a correlative study for both silver cluster fluorescence and electric field induced second-harmonic generation resulting from fs DLW in silver-containing phosphate glasses where the associated electric field was proven to be a key parameter for silver clustering^27^.

To gain a deeper understanding of the underlying physics and associated chemical changes, we have analyzed the laser tracks as an attempt to explain the origin of the obtained high birefringence. In this article, we report on the correlative microscopy studies of Raman, luminescence and polarized second harmonic generation responses that resulted from femtosecond laser irradiation in our zinc gallo-germanate glass. Using micro-Raman and micro-luminescence, we show respectively disruption of the network and generation of defects. Then, we depict oriented spatial distributions of SHG with increasing laser pulse energy. Such work provides a new interpretation of the mechanisms at play post to direct laser writing in BGG glasses.

## Material and methods

### Glass synthesis

Glasses were prepared by the traditional melt-quenching technique. The following raw materials: barium carbonate BaCO_3_ (Fox Chemicals, 99.99%), gallium oxide Ga_2_O_3_ (Fox Chemicals, 99.999%), germanium oxide GeO_2_ (Fox Chemicals, 99.999%) and zinc oxide ZnO (Alfa Aesar, 99.999%) were weighed in the required proportions to prepare a batch of 7 g. Then the powders were mixed in a platinum crucible and heated up to 1550 °C for 1 h. The melted glass was formed by quenching in ambient air. Glass was then annealed at *T*_*g*_ − 40 °C for 4 h and slowly cooled down to room temperature to reduce residual mechanical stress. Finally, it was cut and polished on both parallel faces for fs-laser irradiation.

### Fs laser irradiation

A commercial Yb-doped fiber amplifier fs laser (Satsuma, Amplitude Systèmes Ltd. Pessac, France) delivers the pulses of 1030 nm at a pulse duration of 250 fs with a typical average power up to 10 W. The beam was focused to 500 μm depth beneath the surface of each sample using 0.6 numerical aperture (NA) aspheric lens (estimated beam waist ω ~ 1.5 μm) and pulse repetition rate 100 kHz. Furthermore, a series of lines of 2.5 mm length, were inscribed with a laser scanning speed of 10 μm/s (it meant 10,000 pulses/μm at 100 kHz) for avoiding any diffraction effect. Moreover, the pulse energy was varied from 0.3 to 1.6 μJ and the laser polarizations were in Xx and Xy configurations (X, laser writing orientation and x, y so-called laser polarization direction 0° and 90°, respectively). Polarization-dependent birefringence, characteristic to the presence of nanogratings, were confirmed by field-emission gun scanning electron microscope (FEG-SEM) and the results are published elsewhere^19^. Pulse energies 0.3 and 0.5 µJ correspond to type I regime, while 0.8, 1.2 and 1.6 correspond to type II regime^19^.

### Characterization of modified regions

**Figure 1:**
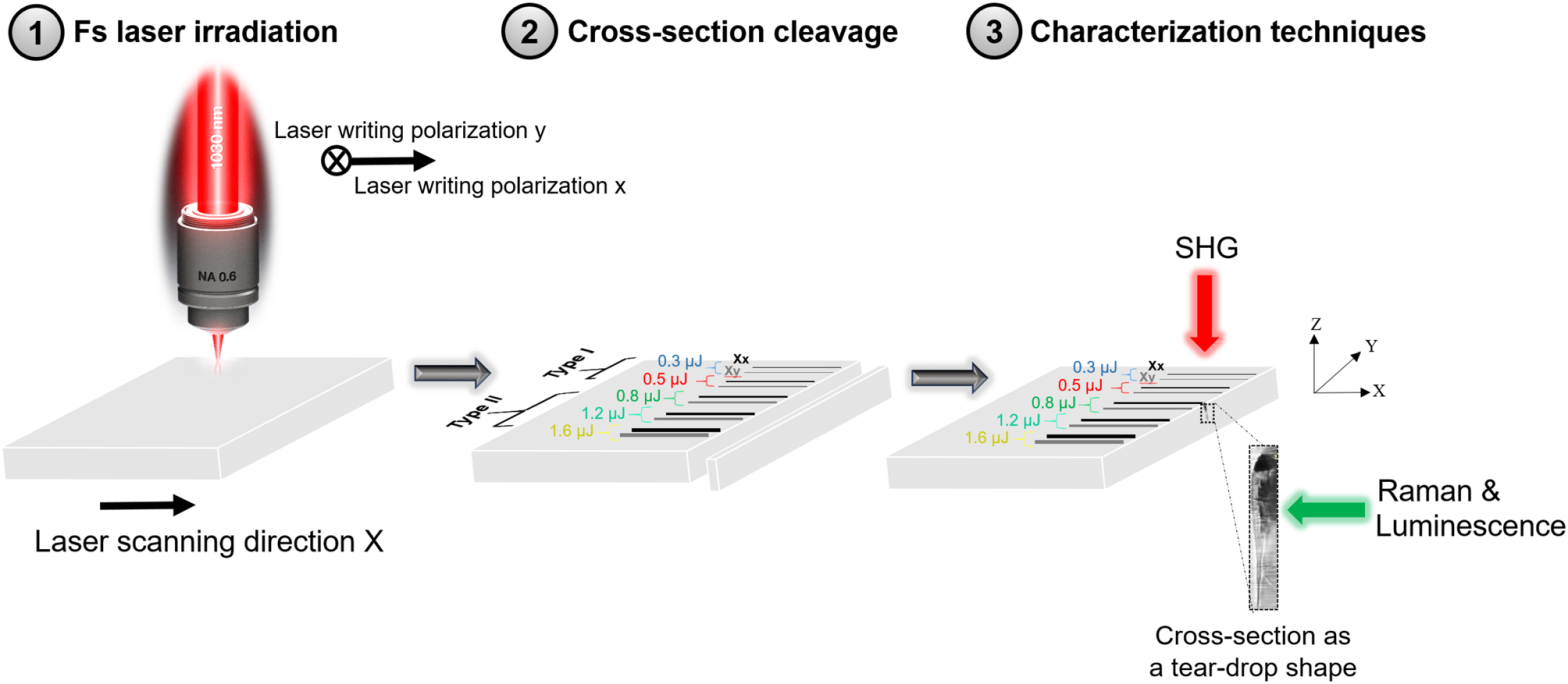
(**1**) Sketch of femtosecond laser writing in 8%ZnO-BGG bulk glass where the laser propagation direction is Z and the laser scanning direction is X. (**2**) Laser traces written by femtosecond laser pulses with pulse energies 0.3, 0.5 µJ corresponding to type I; and 0.8, 1.2, 1.6 µJ corresponding to type II for both writing polarizations x (represented by dark-colored line) and y (represented by light-colored line). The laser scanning direction X corresponds to a direction perpendicular to the beam propagation axis. Then, cleavage of the sample is done to observe the cross-section of the laser tracks appearing in the form of a teardrop-shaped structure. (**3**) Characterization techniques: SHG measurements were done on the top of laser tracks, while Raman and luminescence measurements were done on the cleaved part.

To further study the impact of laser irradiation on the sample, it is useful to give a notion about the sample preparation prior to characterization as can be illustrated in Fig. 1. After FLDW in the glass, SHG measurements were carried out on the surface of the laser tracks. However, to characterize them using Raman and luminescence, the sample was cleaved to access the cross-section of the laser tracks.

Micro-Raman spectra were recorded with a confocal micro-Raman spectrometer LabRAM HR Evolution (Horiba Jobin Yvon) equipped with a Synapse CCD detector using a 532 nm radiation from a diode pumped solid state laser (output power = 20 mW). The incident laser beam was focused onto the sample through a microscope with a 100 × objective (NA = 0.9, Olympus) with acquisition data recorded between 200 and 2000 cm^−1^. Scattered light was dispersed by 1200 grooves/mm holographic grating system. Raman spectra have been corrected by the Bose–Einstein factor. All normalization has been done to the integrated area of the full Raman response.

Micro-luminescence was realized with a LABRAM 800-HR spectrophotometer (Horiba Jobin–Yvon) and a 50 × microscope objective (NA 0.75) using a 405 nm excitation wavelength. Micro-luminescence spectra were recorded thanks to a thermoelectric cooled CCD Camera (Synapse Model 354308). We conducted the measurements twice, first measurements using the 150 grooves/mm grating which is blazed at 500 nm and second time using the 300 grooves/mm grating which is blazed at 1000 nm. The experimental spectra were corrected using a correction function created from luminescence measurements of a calibrated lamp.

Polarized SHG images were recorded at room temperature on a custom-built scanning SHG microscope developed at the ISM lab^28,29^. The excitation source used was a 1064 nm picosecond laser (Leukos Opera), which delivers 50 ps pulses at a repetition rate of 1 MHz. The laser incident light was set at an average power of 450 mW and focused into the probed structures inscribed at ~ 300 µm beneath the surface with a near-IR 20 × objective (Mitutoyo M-PLAN APO, NA 0.4). The resultant SHG signal at 532 nm was collected in the backward direction (epifluorescence mode) using a photomultiplier tube. Polarized SHG images of 20 × 20 μm^2^ where recorded with a spatial resolution of 0.25 μm along the x and y directions. The longitudinal resolution was estimated to be *ca* 5–10 µm since the setup is not confocal by construction.

## Results

### Micro-Raman spectroscopy

**Figure 2:**
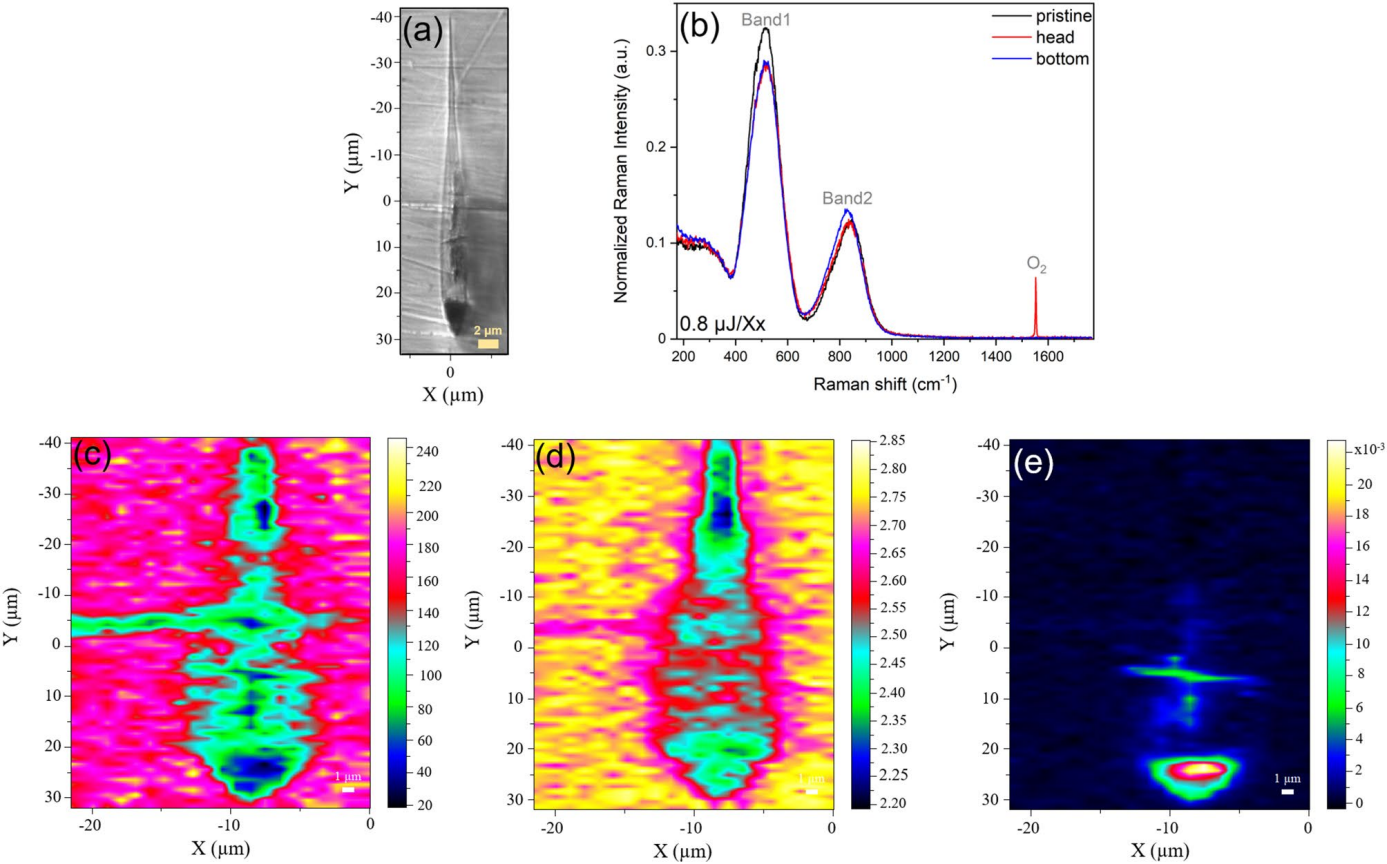
(**a**) Optical microscopy image of the cross-section of the laser track with a pulse energy of 0.8 µJ in Xx configuration. (**b**) Raman spectra normalized to area for three different cases: pristine glass, head and bottom of the teardrop-shaped structure. (**c**) Mapping of the Raman intensity retrieved by integrating the full Raman intensity from 177 to 1580 cm^−1^ without normalization. (**d**) Mapping of the ratio Band1/Band2 retrieved by integrating the normalized spectral region from 380 to 670 cm^−1^ (Band1) and from 670 to 1000 cm^−1^ (Band2). (**e**) Molecular oxygen distribution (peak at 1555 cm^−1^). The laser writing parameters were 1030 nm, 10 µm/s, 100 kHz, 0.6 NA, 500 µm depth.

In order to investigate the structural changes occurring in the modified regions, micro-Raman measurements were performed on the cross-section of the laser tracks, i.e. the teardrop-shaped structure. To begin with, we show in Fig. 2a the cross section of the laser track corresponding to a pulse energy of 0.8 µJ in the Xx configuration.

Figure 2b shows normalized Raman spectra of three different regions for the Xx writing configuration with a pulse energy of 0.8 µJ: the pristine glass, head and tail parts of the teardrop-shaped structure as shown in the optical microscopy image presented in Fig. 2a.

First, we focus on the vibrational spectra of the unmodified or pristine glass which can be decomposed into low (200–400 cm^−1^), intermediate (400–600 cm^−1^) and high frequency regions (600−1000 cm^−1^). The low and intermediate frequency envelopes can be attributed to bending modes involving mainly T^4^–O–T^4^ bridges with T being either gallium or germanium; the brackets refer to the coordination number of the atoms^30–32^. These bending motions centered at 515 and 300 cm^−1^ can be attributed to oxygen motions, respectively, in the plane and out of the plane formed by a bent T–O–T bridges. These modes are due to the 3D network and are coupled to stretching^30^. While the high-frequency component can be assigned to symmetric and antisymmetric stretching features of Ga–O^−^–Ge bridges^30^ as well as localized stretching modes of Ge–O links involving non-bridging oxygens (NBO), mainly *Q*^2^ and *Q*^3^ germanate units^33,34^ where *Q* represents structural units (here tetrahedral ones) and the exponents correspond to the number of bridging oxygens between two glass former cations (Ge or Ga).

Regarding the head and the tail parts constituting the modified region, one can notice a decrease of Band1 for both, and an increase of Band2 only for the tail part. The third prominent feature is the spectral line centered at 1555 cm^−1^ corresponding to the symmetric stretching of molecular oxygen.

An example of three Raman images of a teardrop-shaped structure is shown in Fig. 2c,d,e. In Fig. 2c, the first map represents the integration of the spectral region from 177 to 1580 cm^−1^ without normalization corresponding to variations of the Raman response of the glass upon irradiation. One can see that the Raman intensity is lower in the modified region compared to the unmodified one by a difference of one order of magnitude. This indicates that the material density has decreased in the teardrop-shaped structure, where one can suspect glass expansion (a permanent positive strain), which can result in turn into a negative elastic strain in and around the irradiated area confirmed by polarized microscopy observations (data not shown here). In Fig. 2d, the second map depicts the mapping of the ratio of Band1/Band2 by integrating the normalized spectral region from 380 to 670 cm^−1^ (Band1) and from 670 to 1000 cm^−1^ (Band2). One can notice a decrease in the relative Raman intensity in the teardrop-shaped structure which denotes breaking of the 3D glass network. Both abovementioned changes occur within the entire teardrop-shaped structure. The last image (Fig. 2e) presents the molecular O_2_ distribution. We clearly see that the oxygen is located only on the head where the nanogratings are formed^19^.

**Figure 3:**
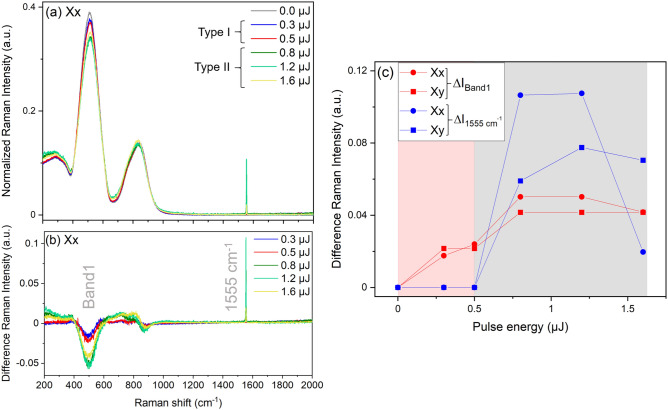
(**a**) Normalized Raman spectra of the structural changes on the head of the teardrop-shaped structure at different laser pulse energies in Xx configurations. The Raman spectrum of the initial glass (before) is also shown. (**b**) Raman intensity difference spectra before and after laser irradiation at different pulse energies in the Xx configuration. (**c**) Plot of the difference Raman intensity of Band1 at 511 cm^−1^ (ΔI_Band1_, in absolute values) and the 1555 cm^−1^ band ($\Delta {\text{I}}_{1555\text{cm}^{ - 1}}$) shown in Fig. 3b with an error of ±5% as a function of the pulse energy in both Xx and Xy configurations. The laser writing parameters were 1030 nm, 10 µm/s, 100 kHz, 0.6 NA, 500 µm depth.

Hereafter, we concentrate on the description of the induced structural modifications on the head part as a function of the laser pulse energy. The Raman spectra of all the pulse energies have been normalized to area and the results are presented in Fig. 3. With increasing the laser pulse energy, evolution could be detected in the relative intensity of the abovementioned Raman modes centered at 300 cm^−1^, 515 cm^−1^ and 830 cm^−1^ between the modified regions and the glass before irradiation. The changes, which are the same as described in Fig. 2, could be better seen in the Raman intensity difference spectra presented in Fig. 3b. One can notice (1) a relative increase in the intensity of the band at 300 cm^−1^, (2) a relative decrease in Band 1 at 515 cm^−1^, (3) a relative increase in intensity together with a red shift of ~ 9 cm^−1^ of the band at 830 cm^−1^ for the maximum pulse energy from that before irradiation and (4) a high-wave number peak at 1555 cm^−1^, attributed to trapped molecular O_2_^5^, was observed for pulse energies above 0.5 µJ. This signifies that O_2_ molecules were locally generated, coherent with observations of nanoporosity revealed by SEM in Ref.^19^. Considering this fact, the voids can be produced as a result of the decomposition of the glass network as in the case of GeO_2_^35^ or SiO_2_^9^. The degree of the structural change depending on the pulse energy can be better elucidated by plotting the difference Raman intensity of Band1 at 511 cm^−1^ (ΔI_Band1_) in absolute values and the 1555 cm^−1^ band ($\Delta {\text{I}}_{1555\text{cm}^{ - 1}}$) as a function of the laser pulse energy as shown in Fig. 3c. When the pulse energy is lower or equal to 0.5 µJ, in other words as long as we are in type I regime, ΔI_Band1_ decreases slightly with the increase of the pulse energy. ΔI_Band1_ continues to decrease but steeper than before once we are in type II regime, up to 0.8 µJ then remain constant at higher energies. On the other hand, the 1555 cm^−1^ peak, which is null in type I, increases rapidly after 0.5 µJ and stabilizes at laser pulse energies greater than 0.8 µJ. The decrease of O_2_ peak intensity for the pulse energy of 1.6 µJ in Xx configuration in Fig. 3c does not signify it is generated in low quantity due to the fact that one probes the post-mortem events, where O_2_ molecules could have escaped or re-distributed. Overall, the decrease of the 515 cm^−1^ band and the subsequent increase of the 1555 cm^−1^ peak indicate the breakage of the T–O–T bridges. These observations of the structural changes appear to be the same in both the Xx and Xy configurations as presented in Fig. 3c. These changes indicate the rigidity of the glass network toward femtosecond laser writing in which the limitation of the cationic mobility is the primordial factor.

### Micro-luminescence

**Figure 4:**
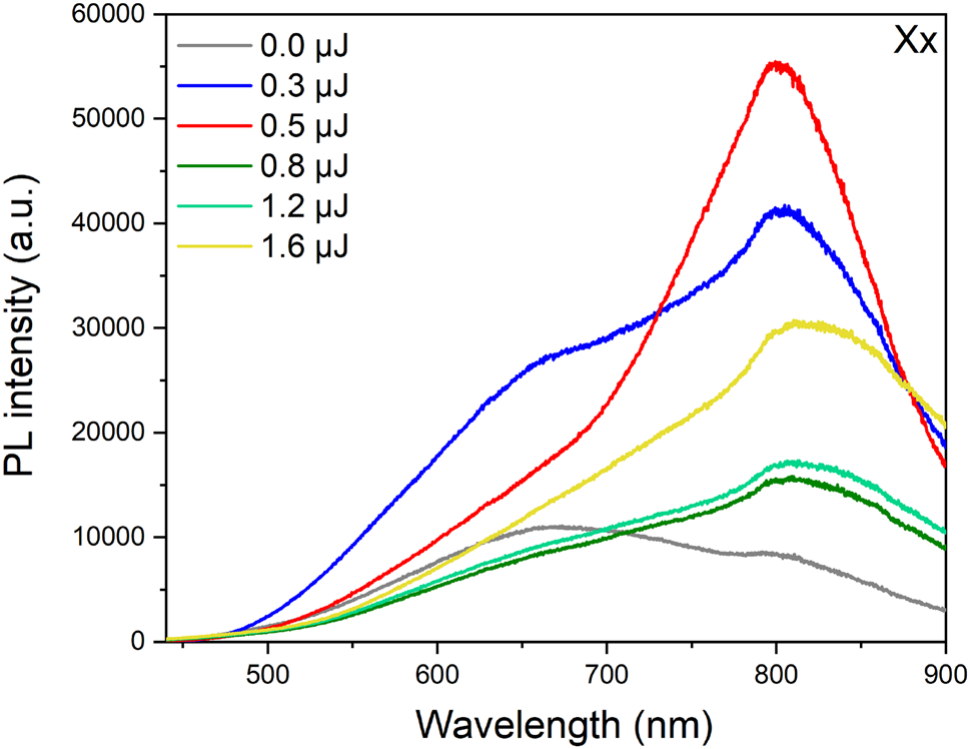
Micro-luminescence spectroscopy with an excitation at 405 nm of the unmodified and modified regions at different pulse energies in the Xx configuration. The laser writing parameters were 1030 nm, 10 µm/s, 100 kHz, 0.6 NA, 500 µm depth.

The micro-luminescence spectroscopy, using a confocal microscope, has been conducted on the unmodified and modified regions for an excitation at 405 nm. We did mapping of luminescence along the length of the teardrop-shaped structure and we found out that the luminescence intensity is maximum at the top of the teardrop-shaped structure. On Fig. 4 is depicted the spectral features collected at the cross-section of the tracks. We were very careful when applying the correction file to the spectra. For that sake, we did the measurements twice using two gratings, the 300 grooves/mm and 150 grooves/mm. A wide emission band is observed between ~ 450 and ~ 900 nm for both the pristine and the irradiated glass. However, depending on the pulse energy, the emission maximum wavelengths and intensity are varying. In the unmodified region, the maximum emission wavelength is around 660 nm with a shoulder at around 800 nm, while in the modified regions the maximum emission is located around 800 nm with a left shoulder at around 660 nm. With increasing the pulse energy, the infrared band becomes more important. Moving from type I to type II modifications, the emission maximum wavelength shifts from ~ 818 nm for a pulse energy of 0.8 µJ to a higher wavelength of ~ 830 nm for 1.6 µJ. Regarding the intensity, there is a clear increase in type I regime, then it substantially decreases in type II modifications. This decrease could be due to the presence of less defect centers or the appearance of porosity in type II regime that could anneal the electron and hole traps and thus affect the photoluminescence. Moreover, we have to take into account the volume of the probed zone. To sum up, there are spectral changes regarding the global intensity but it is difficult to draw from it definite conclusions.

Regarding the spectral assignments, the weak broad band observed for the unmodified glass is similar to bands generally characteristic of defects in oxides, as oxygen deficiency related defects or oxygen dangling bonds. Herein, the band around 660 nm which becomes important in the modified regions is attributed to NBOHC photoluminescence. Its structure is constituted by a dangling bond of an oxygen linked to a germanium atom by a single bond^23,24^. Upon irradiation, the most widely accepted picture of the NBOHC generation is related to the free carrier trapping, after plasma generation, as the self-trapped excitons, leading to stress and consequently breakage of Ge–O bonds. The broken bonds result in the formation of GeE’ (a neutral color center defect where a Ge is linked to three oxygens and the fourth link is replaced by a lone pair of electrons) and Ge-NBOHC, and could result in the release of oxygen (at the origin of trapped molecular O_2_) leading to a germanium oxygen deficient center ODC(II) as it is the case in SiO_2_^21^. It is important to mention that we have not observed Ge-ODC(II) or Germanium Lone Pair Center (GPLC) at ~ 400 nm even by changing the excitation wavelength to 325 nm, due to the fact that our glass matrix has a maximum absorption at this wavelength^23^. Regarding the broad intense band at ~ 800 nm, there is no clear assignment for it in the literature, nonetheless it could be related to reduced species of germanium that have been attributed by Ref.^36^.

### Polarized second harmonic imaging

**Figure 5:**
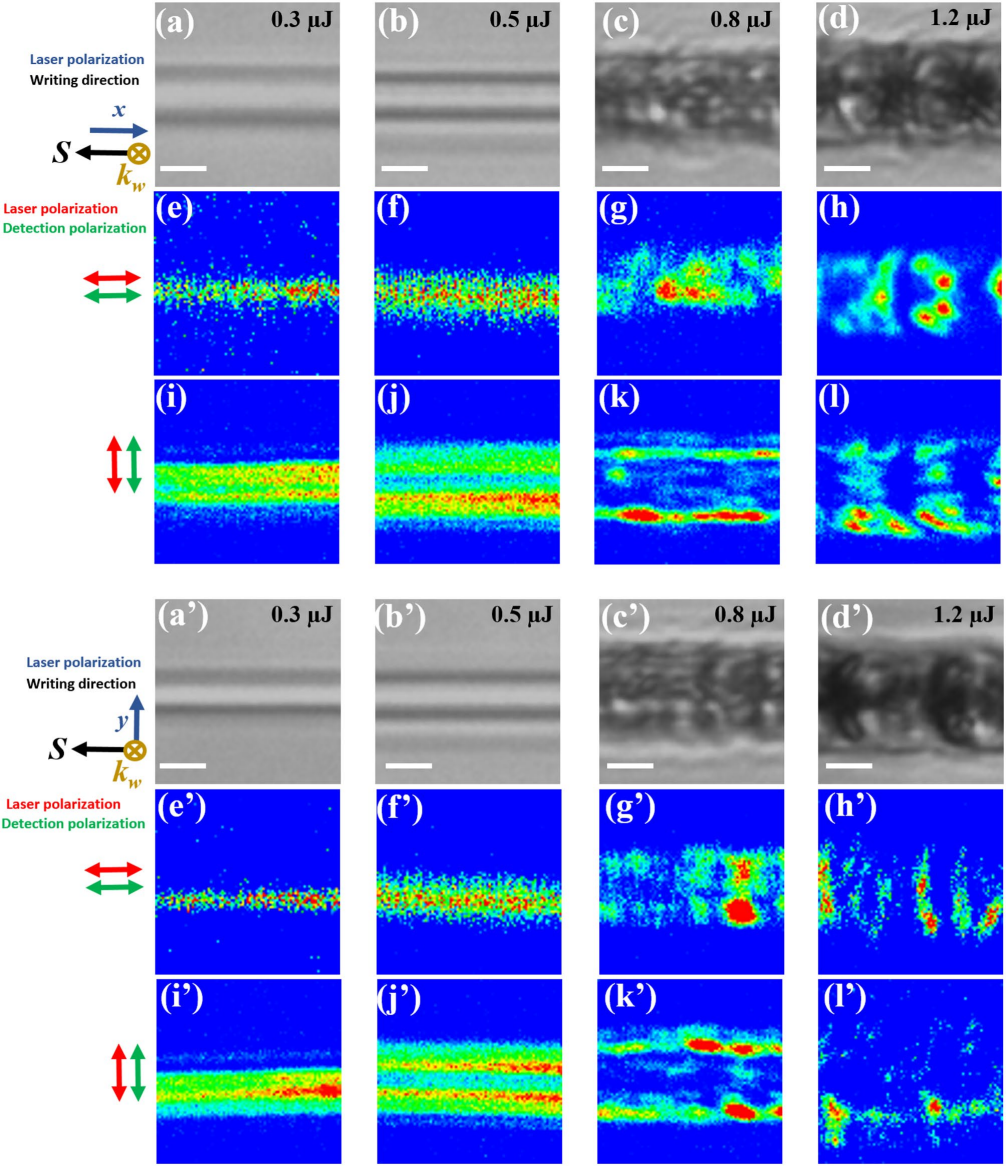
Optical microscopy images of laser tracks by the femtosecond laser pulses with pulse energies of 0.3 µJ (**a**, **a’**) or 0.5 µJ (**b**, **b’**) or 0.8 µJ (**c**, **c’**) or 1.2 µJ (**d**, **d’**) for each writing laser polarization; (**a**, **b**, **c**, **d**) correspond to Xx configuration and (**a’**, **b’**, **c’**, **d’**) to Xy configuration in the 8%ZnO-BGG sample. (**e**, **f**, **g**, **h**) represents the micro-SHG mapping I_XX_ of laser tracks (**a**, **b**, **c**, **d**). (**i**, **j**, **k**, **l**) represents the micro-SHG mapping I_YY_ of laser tracks (**a, b, c, d**). (**e’**, **f’**, **g’**, **h’**) represents the micro-SHG mapping I_XX_ of laser tracks (**a’**, **b’**, **c’**, **d’**). (**i’**, **j’**, **k’**, **l’**) represents the micro-SHG mapping I_YY_ of laser tracks (**a’**, **b’**, **c’**, **d’**). Symbol of *k*_*w*_ indicates the laser propagation direction and *S* the writing direction. Scale bars in white color are 5 µm. The color code is optimized to the amplitude of the signal for better observation. The real intensity is provided later in Fig. 6.

We have carried out polarized micro-Second Harmonic Generation (micro-SHG) mapping measurements on the top of the laser tracks as depicted in Fig. 1. Figure 5 shows the spatial and intensity change of the SHG signal as a function of the writing laser pulse energy and polarization. SHG imaging was achieved with two linear state configurations of light polarization. A linear incident polarization oriented either along the X or Y axis and similarly, either the X or Y linear polarization of the returning SHG signals are analyzed. It forms a combination images of SHG intensity (I_XX_, I_YY_, I_XY_ and I_YX_; the first and second indexed letters correspond respectively to the incident light polarization and the SHG analyzed polarization).

Depending on the laser beam polarization and polarization analysis, one can see that the location of the SHG signal across the inscribed lines is different. For lines inscribed with a pulse energy of 0.3 µJ (type I) in both Xx and Xy configurations, one can see a signal in the middle of the laser track for the measured SHG I_XX_ (Fig. 5e,e’). However, for the measured SHG I_YY_, one can observe, without certainty, 2 or 3 aligned signal lines in the middle of the laser track (Fig. 5i,i’). For a higher pulse energy of 0.5 µJ but still in the type I regime, the signal for the measured XX polarization still exists in the middle but is wider (Fig. 5f,f’), while the 2 borders are now well-separated for SHG in I_YY_ polarization (Fig. 5j,j’) for both Xx and Xy configurations. At higher energies, i.e. at 0.8 µJ where we start having type II structures, the material starts to become perturbed as shown in Fig. 5k,k’ and 2 signal lines at the borders of the laser track are still persistent, however they are spatially slightly intermittent for the measured SHG I_YY_. Likewise, for the measured SHG I_XX_ (Fig. 5g,g’), the signal persists in the middle but also in a sporadic manner. SHG structures start to be randomly distributed at higher pulse energies of 1.2 µJ due to the presence of voids for both I_XX_ (Fig. 5h,h’) and I_YY_ (Fig. 5l,l’) polarizations. This behavior is the same in both Xx and Xy laser writing polarizations. It is worth noting that in our measurements we cannot achieve a resolution comparable to the nanograting period (~ 200 nm) which can only give access to the result from a far field. In the case of very high pulse energies of 1.6 µJ, the SHG signal is destroyed due to the formation of hollow structures with larger voids.

Now, as an attempt to figure out the origin of the SHG signal, we have compared the SHG intensities measured in parallel polarization configuration I_XX_ and I_YY_ with the cross-polarized ones I_YX_ and I_XY_ respectively (Fig. S1) for one pulse energy. Figure S1 shows four polarized SHG images of a 20 × 20 µm^2^ wide zone for the 0.8 µJ energy pulse in Xx configuration. The SHG signals are located either in the center or at the borders of the tracks and are highly dependent on both the incident light and the SHG polarization states. For a field induced along x-axis, the χ^(2)^ tensor comprises only 2 non-null terms χ^(2)^_XXX_ and χ^(2)^_XYY_. On the other hand, for a field induced along y-axis, the χ^(2)^ tensor comprises only 2 non-null terms χ^(2)^_YYY_ and χ^(2)^_YXX_. By comparing the SHG intensities in parallel and crossed light polarization configurations, we observe that the χ^(2)^_XXX_ and χ^(2)^_XYY_ terms are maximum in the center and null at the borders along the *x*-axis of the laser track. Similarly, χ^(2)^_YYY_ and χ^(2)^_YXX_ are maximum at the borders and null in the center of the laser track. Moreover, the SHG images probed with the same SHG polarization state shows a high spatial similarity. Therefore, such spatial correspondence allows evaluating the polarization ratios (i.e. I_XX_/I_YX_ and I_YY_/I_XY_), which are found to be ~ 9 for all the SHG active regions. We have to note that these ratios have been calculated using an average method. These observations suggest the following relations between the engraved χ^(2)^ components: χ^(2)^_XXX_ = 3. χ^(2)^_XYY_ in the center and χ^(2)^_YYY_ = 3. χ^(2)^_YXX_ at the borders of the laser track. The resulting SHG is thus attributed to the charge separation during laser irradiation and traps’ formations leading to an embedded electric and to Electric Field Induced Second Harmonic Generation (EFISH) effect with χ^(2)^ = χ^(3)^. E_stat_, E_stat_ being the static electric field and having two components along x and y^37^. Further, for all the analyzed pulse energies from 0.3 to 1.6 µJ in both Xx and Xy configurations, the polarization ratio I_YY_/I_XY_ ≈ 9 is observed. However, for the polarization ratio I_XX_/I_YX_, it is difficult to estimate it because both intensities I_XX_ and I_YX_ polarization states are too weak. Hence, in this case, it is difficult to say if the SHG signal is of an electro-optic origin or not.

**Figure 6:**
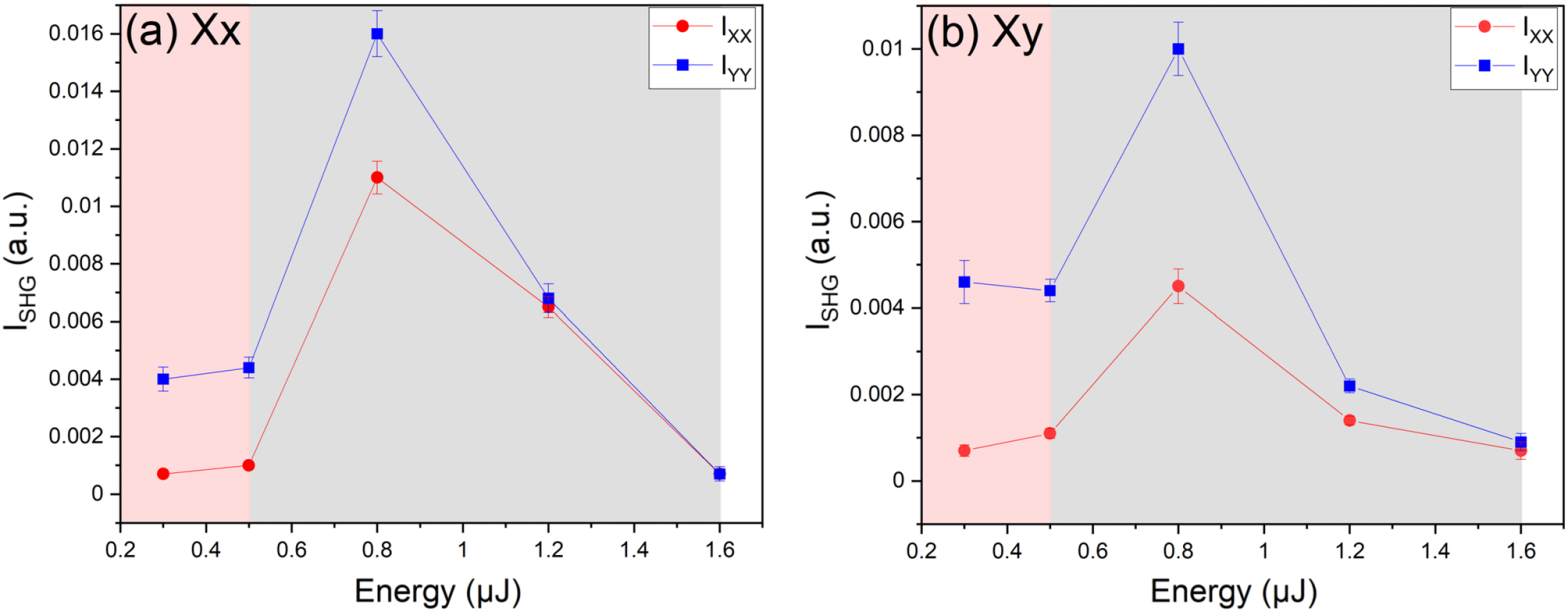
SHG intensity of the laser tracks in the (**a**) Xx and (**b**) Xy configuration as a function of the laser pulse energy in the 8%ZnO-BGG sample. Red traces represent the collected second harmonic I_XX_, blue traces represent the collected harmonic I_YY_. The shaded pale red zones correspond to type I regime and the shaded grey one to the type II regime.

Now to quantitatively study the SHG signal, we plot in Fig. 6 the intensity of the micro-SHG results for the collected second harmonic I_XX_ and I_YY_ versus the laser pulse energy in both Xx and Xy configurations. We have to note that the SHG intensity values have been calculated using an average method. First, we observe that the orientation of the electric field, at least one can say for the 0.8 µJ pulse energy, is mostly perpendicular to the inscribed lines because the collected second harmonic I_YY_ is always stronger than I_XX_. As a second remark, both graphs show the same trend. The SHG intensity remains quite low within type I regime whereas it increases within the type II domain to achieve a maximum at 0.8 µJ. Then it decreases with further energy increase, probably due to more disordered nanogratings as well as damage and charges losses. Furthermore, if we calculate the SHG anisotropy factor Δ i.e. Δ = (I_YY_—I_XX_) / (I_YY_ + I_XX_)), one can see a factor 2 when comparing the two writing configurations namely Xx and Xy within type II regime. Indeed when the writing polarization is along X, Δ = 0.19 whereas Δ = 0.38 for Y writing polarization for a pulse energy of 0.8 µJ. In contrast, within type I regime (typ. 0.3 µJ) the SHG anisotropy factor remains the same whatever the writing polarization may be Δ = 0.71 + /− 0.02. Therefore, this difference likely suggests that the writing configuration plays a role in the SHG relative field strength along the X- and Y-axis in the type II regime.

We have checked for the pulse energy that gave the highest SHG signal for the Xx writing configuration the Raman response in all polarizations: VV, HH, VH and HV respectively corresponding to ZZ, YY, ZY and YZ. The resolution in the x–y plane which is defined by the focal laser spot size corresponds to a value of 1.3 µm. We see in Fig. S2 that the polarized Raman VV = HH and VH = HV. This signifies that Raman measurements of the laser tracks do not evidence anisotropy for this laser pulse energy (0.8 µJ). We have to note that this observation does not depend on the position in the trace.

## Discussion

Comparing the trends observed in Figs. 3c and 6 with increasing the pulse energy, it appears that the SHG results and the vibrational structural changes observed in Raman as well as the point defects formation (seen through photo-luminescence shown Fig. 4) give complementary information related to the fs laser-matter interaction mechanisms.

Within type II regime, by combining the global Raman intensity map with the determination of the slow/fast axes orientation of the stress-induced birefringence, we can deduce the presence of a local volume expansion correlated with the formation of nanoporous glass within nanolayers^38^. This net volume expansion is further confirmed by the appearance of a compressive stress field as it is the case for nanogratings formation in SiO_2_^38,39^. In addition, Raman shows a clear evidence of T–O–T bonds breaking in both the head and the tail of the tear-shaped laser tracks highlighted by the decrease of the band peaking at 515 cm^−1^ related to the 3D glass network. Such phenomenon originates from two different reasons. At first, this occurs in the head of laser tracks due to the nanograting formation associated with ODC and O_2_ formation. But this also happens in the tail due to ion migration resulting in an increase in intensity of the high frequency band corresponding to NBO on germanate units. Therefore, we have mostly glass expansion throughout the tear-shaped structure and the Raman map likely suggests a chemical migration (Zn^2+^ or Ba^2+^) towards the tail part of the modified region as already proposed^40–45^.

The appearance of molecular O_2_ in Raman measurements tends to coincide with the maximum of SHG in type II regime at 0.8 µJ. On the contrary, luminescence appears to be maximum in type I regime. After that, it decreases within type II regime together with SHG, which starts to decrease after 0.8 µJ, while the Raman changes remain constant. Thus, regarding the different phenomena, one can recognize at least two regimes depending on the pulse energy.

Explicitly, in the first regime up to ~ 0.5 µJ, the main occurrence is the important generation of charge traps clearly evidenced by the luminescence increase, and simultaneously separation of charges and formation of a static field as confirmed by the EFISH origin of the second order optical response. These findings are, on one hand, in accordance with the work reported on silver-containing phosphate glasses in which the first phenomenon that takes place after femtosecond laser irradiation is the SHG^27^, and on the other hand with previously reported work on germanosilicates where SHG is associated with photo-induced defects^25,26^. Yet, we also see disruption of glass former bonds in Raman as stated above, revealing a chemical change. Indeed, for the lowest pulse energies, electrons-holes pairs are first produced at the center where the intensity is maximum, followed by a fast thermalization, migration of free electrons towards low intensity zone and traps formation. This process will vanish the initial spatial charge neutrality of the electron–hole plasma inside the focal spot and creates an ambipolar electric field. This phenomenon is analog to the situation reported for silver-containing phosphate glass after femtosecond direct laser writing in which the whole process of charge separation gives rise to the depletion of silver ions in the center and the formation of silver clusters after electron trapping on the periphery of the laser beam^46^. *Thus, our results are in agreement with previous studies pointing out the occurrence of an electric field following femtosecond laser direct writing.*

Within type II regime e.g. at ~ 0.8 µJ, both SHG and Raman modifications attain maximum intensities. For example the appearance of molecular O_2_ in Raman measurements tends to coincide with the maximum of SHG in type II regime at 0.8 µJ. Then up to ~ 1.6 µJ, we do not distinguish further Raman evolution which could be related to the spatial resolution that does not allow to resolve the nanograting i.e. strongly affected and “unaffected” areas, so one probes an average volume. Therefore, we could underestimate the structural changes. Nonetheless, the main occurrence is definitely a strong chemical change and re-organization of the glass network proved by the continuous production of O_2_ traducing bonds breaking. In addition, the emission band at around 818 nm attributed to GeO_2_ reduced species diminishes from ~ 0.5 to ~ 1.6 µJ, which could be related to the reorganization of the glass network and the reactivity of O_2_ that could annihilate these defects. One can imagine that in some zones where we have ODCs, the excess of O_2_ will compensate them for instance.

In Ref.^21^, the physical origin of the nanogratings in silica was revealed to be made of quasi-periodic porous nanolayers. The nanopores’ formation was later interpreted through a nanocavitation process^47,48^ coupled to scattered electromagnetic waves interference^49^ thus creating elongated nanopores perpendicularly to the laser polarization. There is no chemical migration in this model but a destabilization of SiO_2_, at the place of high electron density, induced by electron plasma energy transfer to the lattice. However the results in this paper may suggest an alternative and more general pathway, where the plasma imprints charge redistribution due to high electronic mobility and very likely some ionic migration. For example we can clearly observe that the SHG anisotropic factor Δ is strongly impacted by the nanogratings orientation themselves resulting in a much higher Δ when nanolayers are parallel to the scanning direction (i.e. for a Xy writing configuration). In addition, such multi-pulses experiment (typ. 10^4^ pulses/µm) clearly highlight the possibility to induce and maintain a postmortem electric field, along with the induced potentials, which are finally imprinted in the solid. The electrostatic process proposed here is likely of similar nature to the process described in Ref.^50^. Indeed the laser-imprinted electric field (leading to the observed SHG) has to be taken into account in the physico-chemical reaction of nanogratings formation and potentially leading to phase separation. Here, there are two aspects of interest to consider based on the present results. The first is the involvement of the electric field that could drive a phase separation like in Li_2_O-Nb_2_O_5_-SiO_2_ glasses^51^, i.e. a thermodynamic aspect. The second aspect is more related to kinetics, the ionic migration in an electric field that can lead to spatial nanostructuration e.g. a chemical migration (Zn^2+^ or Ba^2+^).

## Conclusions

In this paper, we have reported on an evident correlation between the modification of the glass network, creation of traps and the induced second-order nonlinearity from direct laser writing in the 8%ZnO-BGG glass. At low pulse energies, below 0.5 µJ (type I), there is generation of charge traps resulting in the appearance of luminescence, and stabilization of charges revealed by Second Harmonic Generation. At higher pulse energies, above 0.5 µJ, or in other words, at the intermediate range corresponding to the beginning of type II modifications and the end of type, the glass structure starts to be modified by the destruction of T–O–T bridges in the glass matrix and the generation of O_2_ molecules inside the focal volume, together with the maximum electric field which appears to be dependent on the laser writing polarization. Based on the observed SHG results, we suggest that the organization of NG will be perturbed by such internal electric field (but also likely by an external one). Furthermore, the existence of a post-mortem large electric field photo-induced by fs light excitation open to consider that other effects could be at play like phase separation or electrostriction. The later one may even trigger strain fields and be at the origin of the suggested nanocavitation process thus imprinting porous nanogratings in most oxides glasses. The embedded nanogratings and the dissociated microstructures could be promising materials for multiple practical applications in optical data storage, waveguiding 3D geometric phase optics and more generally for imprinting micro-optical devices especially within a SWaP reduction approach.

## Supplementary Information

Supplementary Information.

## Data Availability

The datasets used and/or analysed during the current study are available from the corresponding author on reasonable request.
