# Supplementary material for: Optical and structural characterization of femtosecond laser written micro-structures in germanate glass
Source: Sci Rep. 2023 Jul 8;13:11050. doi: 10.1038/s41598-023-35730-3 (PMC10329654; doi:10.1038/s41598-023-35730-3)
Supplement: Supplementary file 1 — Supplementary Information. [file 41598_2023_35730_MOESM1_ESM.pdf]

Optical and structural characterization of femtosecond laser written micro-structures in germanate glass

Rayan Zaiter[a*], Matthieu Lancry[b], Alexandre Fargues[a], Frédéric Adamietz[c], Marc Dussauze[c], Vincent Rodriguez[c], Bertrand Poumellec[b], Thierry Cardinal[a]

[a] Institut de Chimie de la Matière Condensée de Bordeaux, Université de Bordeaux, 87 Avenue du Dr Schweitzer, Pessac F-33608, France

[b] Institut de Chimie Moléculaire et des Matériaux d'Orsay/SP2M/MAP, CNRS, Université Paris-Saclay, Orsay 91405, France

[c] Institut des Sciences Moléculaires, UMR 5255, Université de Bordeaux, 351 cours de la Libération, Talence Cedex 33405, France

Supporting information

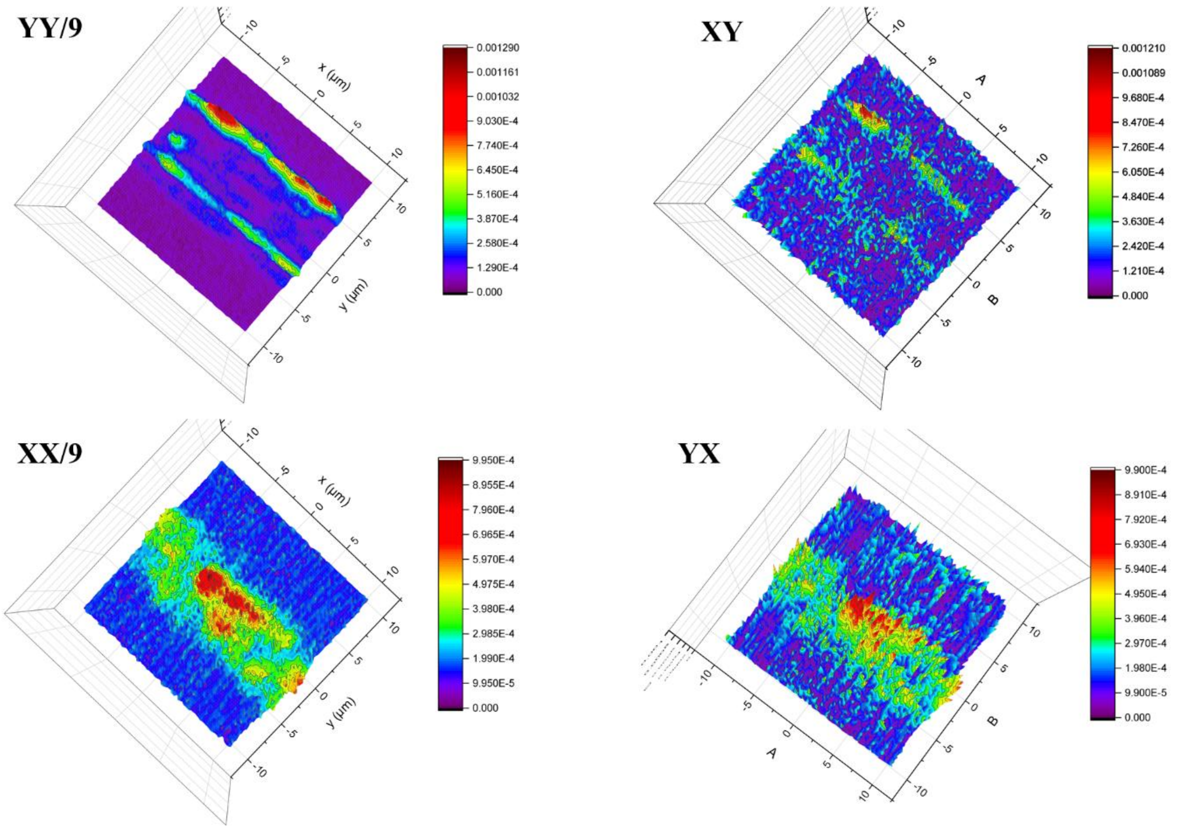

Figure S1. Polarized SHG intensity images of the laser track with a pulse energy of 0.8 µJ in Xx configuration.

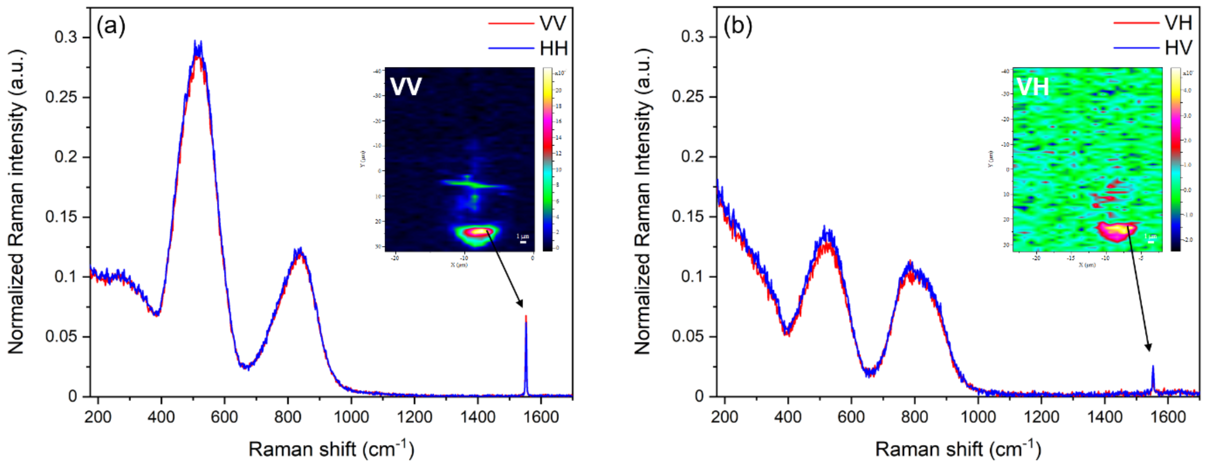

Figure S2. Polarized Raman spectra normalized to area (a) VV and HH, (b) VH and HV for one position referred to by an arrow (corresponding to the head of the tear-shaped structure) for the laser pulse energy of 0.8 µJ in the Xx configuration.

Micro-Raman measurements were recorded in a backscattering mode on a confocal micro-Raman spectrometer HR800 (Horiba/Jobin Yvon). A continuous wave laser operating at 532 nm was focused through a microscope with a 100x objective (NA = 0.5). Raman mapping was realized using a point by point method with a 1 µm step in both the x and the y directions. The measurements were carried out for incident laser polarization and analyzed optical Raman signals vertically, horizontally and cross-polarized. Scattered light was dispersed by 600 grooves/mm holographic grating system.
